# Considering Questions Before Methods in Dementia Research With Competing Events and Causal Goals

**DOI:** 10.1093/aje/kwad090

**Published:** 2023-05-03

**Authors:** L Paloma Rojas-Saunero, Jessica G Young, Vanessa Didelez, M Arfan Ikram, Sonja A Swanson

**Keywords:** competing event, dementia, mortality, survival analysis, time to event

## Abstract

Studying causal exposure effects on dementia is challenging when death is a competing event. Researchers often interpret death as a potential source of bias, although bias cannot be defined or assessed if the causal question is not explicitly specified. Here we discuss 2 possible notions of a causal effect on dementia risk: the “controlled direct effect” and the “total effect.” We provide definitions and discuss the “censoring” assumptions needed for identification in either case and their link to familiar statistical methods. We illustrate concepts in a hypothetical randomized trial on smoking cessation in late midlife, and emulate such a trial using observational data from the Rotterdam Study, the Netherlands, 1990–2015. We estimated a total effect of smoking cessation (compared with continued smoking) on 20-year dementia risk of 2.1 (95% confidence interval: −0.1, 4.2) percentage points and a controlled direct effect of smoking cessation on 20-year dementia risk had death been prevented of −2.7 (95% confidence interval: −6.1, 0.8) percentage points. Our study highlights how analyses corresponding to different causal questions can have different results, here with point estimates on opposite sides of the null. Having a clear causal question in view of the competing event and transparent and explicit assumptions are essential to interpreting results and potential bias.

Dementia researchers face many methodological challenges when addressing causal questions (1), including that individuals at risk of dementia may die of other causes prior to its onset. When dementia is the outcome of interest, death is a competing event because an individual who dies from another cause prior to dementia onset cannot subsequently experience dementia (2). The research field constantly faces counterintuitive results, where exposures that are known to be harmful for mortality risk sometimes seem protective for the risk of dementia, such as smoking (3) or history of cancer (4). Authors have attempted to make sense of these counterintuitive results by naming biases such as “competing risk bias” or “survival bias” (5). However, “bias” cannot be defined or assessed if the causal question or estimand is not explicitly specified.

Despite the fact that methods to “address” competing events have been discussed and developed over several decades (2, 6–9), much of the methodological and applied discussion has been on estimation (10–20) with little emphasis on the estimands. More recent work by Young et al. (21) placed historical estimands from the survival analysis literature in competing events settings within a formal counterfactual framework for causal inference. Specifically, they formalized the interpretation, conditions for identification in real-world studies, and some corresponding statistical methods for estimation.

The goal of this paper is to translate the formal ideas presented in Young et al. (21) for epidemiologists and applied researchers whose understanding might be enhanced through concrete examples, as a crucial first step to aid and encourage more transparent and critical reasoning in scientific studies with competing events and causal goals. Specifically, we will illustrate the choices of the “total effect” and “controlled direct effect” (defined below) through an example where interest lies broadly in a causal effect of smoking cessation (versus not) among smokers on subsequent dementia risk.

To understand the context in which these applications are occurring, and therefore to better understand how these types of challenges are approached in practice, we also conducted a systematic review of longitudinal studies focused (implicitly or explicitly) on causal effects on dementia risk (see Web Appendix 1, Web Figure 1, and Web Table 1 for methods and results, available at https://doi.org/10.1093/aje/kwad090). We found that the vast majority of studies reported cause-specific hazard ratios based on Cox proportional hazards analysis with no explicit interpretation of the effect estimate or justification for its use. The near universal use of such an approach may be due to unsupported and vague guidance in the literature that when the aim of a study is “etiological” (10, 15, 22, 23), the cause-specific hazard ratio is the most appropriate measure or that reporting effect estimates derived from a Cox proportional hazards model somehow amounts to “ignoring” competing events (24). More fundamentally, the majority of these studies did not explicitly report even the number of deaths occurring during the study period. With this backdrop in mind, we now describe the total and controlled direct effect in the context of competing events.

## REASONING ABOUT ASSUMPTIONS AND STATISTICS FOR THE TOTAL AND CONTROLLED DIRECT EFFECT (CASE 1): AN IDEALIZED TRIAL

Suppose investigators were able to conduct an idealized randomized trial such that middle-aged smokers are randomly assigned to a strategy of quitting versus continuing smoking. Dementia onset is rigorously measured through constant screening (therefore minimizing outcome misclassification) and date of death is collected through linkage with municipal records. Further, suppose that in the idealized trial we have complete follow-up (all individuals remain in the study until administrative study end of follow-up or until death) and perfect adherence. Trial participants will be observed to follow different possible event trajectories through the study period: death without developing dementia, dementia onset (some dying after dementia onset), or remaining alive and dementia-free until end of follow-up.

**Figure 1:**
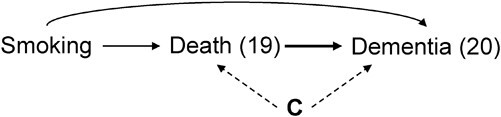
A causal directed acyclic graph representing some key causal features of the data structure. Smoking represents the exposure status (quit smoking vs. continue smoking), Death (19) and Dementia (20) represent indicators of death by 19 years of follow-up and dementia by 20 years of follow-up, respectively. **C** represents a vector of possible shared causes of dementia and death (such as cardiovascular comorbidities), that may or may not be measured. The key relationships are: 1) Smoking may independently affect the risk of both dementia and death over time through different mechanisms; 2) dying over the first 19 years of follow-up (without prior onset of dementia) determines that the indicator of dementia at 20 years of follow-up is zero (the bold arrow representing this key determinism induced by competing events); and 3) dementia and death can have shared causes.

The key challenge of causal inference with competing events is that, for those individuals who died without developing dementia, after the time of death, they cannot subsequently develop dementia. For illustration, consider the causal diagram in Figure 1**,** which represents an underlying data-generating assumption on the key features of this data structure: Smoking represents smoking status at “time zero” (i.e., at the time of randomization), and “Death (19)” and “Dementia (20)” represent indicators of death by 19 years of follow-up and dementia risk by 20 years of follow-up, respectively. By randomization in this study, we know there are no shared causes of smoking and other variables represented in the graph (the only cause of quitting smoking is a “coin flip”). However, we have no such guarantee for death and dementia status over the follow-up and therefore depict shared causes, **C,** of dementia and death (such as cardiovascular comorbidities) that may or may not be measured. The arrows from Smoking to Death (19) and Smoking to Dementia (20) illustrate that smoking may affect both dementia and death through different mechanisms. Smoking may also affect **C**, although we have omitted this arrow for simplicity, and it does not affect the assumptions described below. The bold arrow from Death (19) to Dementia (20) represents the key feature of a competing events data structure: An individual who dies by year 19 of follow-up cannot subsequently develop dementia at the next time point. Although we present death and dementia at years 19 and 20, respectively, the causal diagram could be expanded to include prior assessments, but this simplified causal diagram is sufficient for our consideration of the different causal questions.

Before moving forward to the next section, we note that the historic survival-analysis terminology classifies this structure as “semicompeting events” since death is a competing event for dementia but not the other way around (25). Although several authors consider semicompeting events as a different problem (13, 14), this different designation is unnecessary when we begin with causal questions rather than statistics. That is, when the study event of interest is subject to competing events (again, meaning there exist events that determine that the event of interest cannot subsequently occur) the key considerations for causal inference (defining a causal question and reasoning about identification, which are the drivers of choosing an appropriate analytical approach) are unchanged regardless of whether this target event is nonterminal (such as dementia) or terminal (such as a particular cause of death) (26–28).

### Two different notions of a causal effect on dementia: the total and controlled direct effect

In this section we outline the estimands that represent the total and the controlled direct effect. Formal counterfactual notation is available at Young et al. (21).

The total effect can be quantified by the answer to the following question: What would the difference in dementia risk by 20-year follow-up be had all individuals in the study population quit smoking versus, instead, had all individuals continued smoking? This dementia risk is an example of a “cause-specific cumulative incidence” or “crude risk” in the traditional survival analysis literature (2, 29). The total effect captures all pathways by which exposure affects dementia, which include both a direct effect on dementia (Smoking 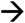 Dementia (20)) and indirect of smoking via mortality (Smoking 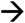 Death (19) 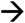 Dementia (20)), as observed in the causal diagram in Figure 1. This indirect effect is necessarily protective since participants who die due to smoking at an earlier time point are “protected” from developing dementia. This “pathological mediation” structure gives the total effect a potentially problematic interpretation, since smoking cessation may increase the risk of dementia but primarily or solely because it delays death. Thus, interpretation of the total effect should be cautious when there may exist an arrow between the exposure and competing event. Empirical support for this arrow might be obtained by also estimating the total effect of smoking cessation on all-cause mortality but should also be considered in light of subject matter knowledge.

Alternatively, a direct effect of smoking on the risk of dementia (that does not also capture the pathways mediated by death) may be of interest. There are multiple ways to define a direct effect (30–32). We present a definition that may lead to familiar statistical methods under certain reasoning about “censoring events” as will be described in the next section: the controlled direct effect. In our example, a question about the controlled direct effect is phrased as: What would the difference in dementia risk by 20-year follow-up be had all individuals in the study population quit smoking and not died throughout the study period versus, instead, had all individuals continued smoking and not died throughout the study period? This dementia risk (under elimination of death) is an example of a “net risk” or “marginal cumulative incidence” (2, 29). This effect captures the direct effect of smoking on dementia because it refers to a hypothetical setting in which somehow death could be eliminated.

The risk differences above both quantify causal effects (represented in counterfactual notation in Table 1) because they both refer to a comparison of outcome distributions under different interventions but in the same individuals. In contrast, while cause-specific hazard ratios are the basis of the majority of analyses in dementia studies, their interpretation as causal effects is problematic, even under the conditions of an ideal trial. Unlike risks, hazards are defined conditional on not yet having had the outcome or competing event. This conditioning means that hazard contrasts do not compare outcomes under different exposures in the same individuals when exposure and other factors affect these events (21, 33–35). Therefore, in dementia studies, cause-specific hazard ratios will not generally have a causal interpretation when exposure affects either dementia or death (directly or indirectly). For this reason, we focus on risks even though most studies in our literature review reported hazard ratios (Web Table 1).

**Table 1:** Comparison of the Total and Controlled Direct Effect of Smoking Cessation on Dementia Diagnosis When Death (a Competing Event for Dementia) Is Present

**Feature**	**Total Effect**	**Controlled Direct Effect**
Estimands for the risk difference^a^	$\Pr\! \left[{Y}_{k+1}^{a=1}=1\right]-\Pr\! \left[{Y}_{k+1}^{a=0}=1\right]$	$\Pr\! \left[{Y}_{k+1}^{a=1,\bar{d}=\bar{0}}=1\right]-\Pr\! \left[{Y}_{k+1}^{a=0,\bar{d}=\bar{0}}=1\right]$
Exchangeability assumption needed for death (competing events)?^b^	Not needed	Needed. Requires that at each follow-up time, conditional on the measured past, death is independent of future counterfactual outcomes had everyone followed $A=a$ and death was eliminated.
Positivity assumption needed for death (competing events)?^b^	Not needed	Needed. Requires that for any possibly observed level of exposure and covariate history among those remaining uncensored (alive) and free of dementia diagnosis through *k*, some individuals continue to remain alive through *k* + 1.
Consistency assumption needed for death (competing events)?^b^	Not needed	Needed. Requires that an intervention that “eliminates death (competing events)” is well-defined.
Interpretation	The effect captures all pathways by which exposure affects dementia, which may include both direct and indirect exposure effects outside and through the exposure’s effect on mortality	Direct effect on dementia not via death, referring to scenario where death has (somehow) been eliminated.

^a^ ${{Y}}_{k+1}^a$ denotes counterfactual dementia diagnosis status by *k* + 1 under exposure level $a$. ${Y}_{k+1}^{a,\bar{d}=\bar{0}}$ denotes counterfactual dementia diagnosis status by *k* + 1 under exposure level $a$ and (somehow) eliminating the competing event such that $\bar{d}=\bar{0}$.

^b^ See Web Appendix 2 for fuller discussion.

In sum, there is no single way to define “the” causal effect on dementia when deaths occur. Choosing to study the total effect, the controlled direct effect, both, and/or other alternative estimands as the causal effect of interest should be done on a case-by-case basis. Furthermore, presenting information on the total exposure effect on all-cause mortality may complement any such estimand.

### Identifying the total effect and the controlled direct effect on dementia

Now that our causal effects have been stated, in this section we discuss the assumptions that help us connect our causal quantity of interest to observable data (i.e., identification). In the idealized trial we considered above, particularly with complete adherence and no loss to follow-up, the total effect can be identified under conditions that are expected to hold by design. Namely, the idealized trial implies we expect consistency, positivity, and exchangeability with respect to the exposure (36, 37).

In contrast, these conditions are not sufficient to identify the controlled direct effect of smoking cessation on the risk of dementia. In Figure 1, we observe the noncausal path between death and dementia through their shared cause **C**, Dementia (20) 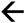
**C**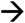 Death (19). Death is a form of censoring for this question, a type of missingness in the outcome of interest (21). When an individual dies prior to dementia onset, dementia onset “under elimination of death” is missing for that individual. While many researchers equate “death” with “censoring” by default, these terms are not synonymous: Death is only a type of censoring (leading to missingness of the dementia outcome) when the question of interest is about outcomes “under elimination of death.” This illustrates that, what constitutes censoring depends on the question of interest, and when censoring is present, we need additional exchangeability, positivity, and consistency conditions with respect to censoring (21). Thus, even in our above-described trial with randomized smoking cessation, we need to measure and adjust for **C** to identify the controlled direct effect.

Figure 1 is consistent with a conditional exchangeability assumption for censoring, conditional on shared cause **C** of dementia and death (2, 21, 38, 39). Assuming the absence of shared causes between death and dementia (i.e., assuming the absence of the dotted arrows from **C** to Death (19) and Dementia (20) in Figure 1) coincides with the assumption of unconditional exchangeability for censoring with respect to **C**, analogously related to unconditional “independent censoring.” Unconditional exchangeability for censoring is implausible for nearly all dementia research when censoring includes death, since both events are related to the aging process, while conditional exchangeability assumptions may become more plausible if measuring and adjusting for a rich set of baseline and time-varying shared causes. A summary of the additional identifiability assumptions in respect to death for both questions is presented in Table 1 and outlined in more detail in Web Appendix 2.

Relatedly, loss to follow-up is a form of censoring for total and direct effects. Since mechanisms of loss to follow-up might be related to impaired cognition and dementia, shared causes of attrition and dementia should be measured (40, 41). Further details on censoring, exchangeability assumptions, and graphical identification of both effects, including scenarios with loss to follow-up, can be found in Young et al. (21).

### Statistical methods to estimate the total effect and the controlled direct effect

Now that we have outlined an idealized trial when competing events are present, phrased 2 alternative causal questions, and discussed the assumptions required for identification, we are equipped to consider appropriate statistical methods. In our idealized trial, the total effect can be estimated by simply comparing 2 proportions: the proportion diagnosed with dementia at 20-year follow-up in the “quit smoking” arm versus the proportion diagnosed with dementia at 20-year follow-up in the “do not quit smoking” arm. In both proportions, individuals who die before developing dementia will contribute to the denominator but never to the numerator. Likewise, these quantities can be estimated with the Aalen-Johansen estimator (21, 29), which extends to settings with loss to follow-up.

In contrast, estimation of the controlled direct effect in the ideal trial will yet require covariate adjustment on the shared causes of death and dementia, to satisfy the conditional exchangeability assumption for censoring. To this matter, one basic possibility is to estimate the controlled direct effect by comparing the risk estimates from the complement of a weighted version of the Kaplan-Meier estimator (21), where weights represent the inverse probability of censoring by death conditional on covariates (21, 38, 39, 42, 43). These covariates should be those assumed to ensure the conditional exchangeability assumption for this form of censoring (e.g., the covariates **C** in Figure 1). We point to inverse-probability weighting as one of multiple estimation methods, given its familiarity and straightforward adaptation to traditional statistical methods in any statistical software (42, 43). However, readers should be aware of methods such as the parametric g-formula, which can serve to answer complex questions for time-varying treatment strategies at the expense of more parametric model assumptions, and open-source software is available to estimate the total effect as well as the control direct effect in settings with competing events (44). Likewise, doubly robust extensions can be adapted to estimate either of these estimands with options to incorporate machine learning (45–47).

Last, we can estimate the risk of all-cause mortality using standard methods like the Kaplan-Meier estimator. Recalculation of the time to death should include the period beyond the dementia diagnosis, for those who had dementia over follow-up. In all cases, straightforward extensions exist for covariate adjustment (e.g., by inverse probability weighting) to address loss to follow-up and confounding (21, 41, 48–50). As such, these methods can be used both in randomized trials and observational studies, although our consideration of estimation in an ideal trial helps illuminate the unique feature of competing events.

## REASONING ABOUT ASSUMPTIONS AND STATISTICS FOR THE TOTAL AND CONTROLLED DIRECT EFFECT (CASE 2): APPLICATION TO THE ROTTERDAM STUDY

We now illustrate the estimation of total and controlled direct effects of smoking cessation on dementia using data collected from the Rotterdam Study, a population-based prospective cohort study (51). Our discussion of this example is intended to highlight the features that are shared with the idealized trial above—namely, how we reason about the assumptions and statistics for the total and controlled direct effect in a real data setting with competing events, where other considerations (e.g., baseline confounding, loss to follow-up) are nonetheless present.

Rotterdam Study participants older than 55 years underwent questionnaire administration, physical and clinical examinations, and blood sample collection at baseline (1990–1993) and at follow-up visits from 1993–1995, 1997–1999, 2002–2005, and 2009–2011. Smoking habits were assessed through questionnaires at study entry via self-reported status as “former smoker,” “current smoker,” or “never smoker.” Dementia diagnosis was collected by screening at each visit and through continuous automated linkage with digitized medical records and regional registries. Death certificates were obtained via municipal population registries with complete linkage. Further details are specified in Web Appendix 3. This ascertainment method means the Rotterdam Study has functionally no loss to follow-up with respect to dementia diagnosis and death. The Rotterdam Study has been approved by the medical ethics committee according to the Population Study Act Rotterdam Study, and written informed consent was obtained.

Individuals aged 55–70 years who reported smoking (current or former) and who did not have history of dementia at cohort entry were eligible for the present study. To emulate the trial described previously, we contrast former and current smokers. This contrast has some limitations when viewed as an emulation of the trial; for example, there may be unmeasured confounding, selection bias due to misaligning “time zero” (52, 53), and measurement error (37). A thorough consideration of these other issues would be critical for evaluating the effect size of smoking cessation on dementia risk but go beyond the scope of this exercise. For didactic purposes, we therefore focus our attention on how the competing event of death affects the interpretation, assumptions evoked, and analytical decisions.

## METHODS

To estimate the total effect of smoking cessation on dementia risk, we compared a weighted Aalen-Johannsen estimator in current versus former smokers with weights defined as a product of inverse-probability-of-treatment weights (37) to adjust for the following possible confounders: age, sex, apolipoprotein E ε4 status, and educational attainment. Briefly, the weight for a current smoker is defined as the inverse of the probability of smoking conditional on covariates, and for a former smoker as the inverse of quitting conditional on covariates. We estimated these probabilities with a logistic regression model for smoking as a function of the above-mentioned covariates.

To estimate the controlled direct effect, we compared the complement of a weighted Kaplan-Meier survival estimator in smokers versus former smokers with time indexed in years. The weights in this case are time-varying by follow-up year, defined as a product of the time-fixed weights above and a year-specific inverse-probability-of-censoring-by-death weights. For an individual still alive in year *t*, the time *t* censoring weight is the product of the inverse probability of surviving in each year prior to *t*, conditional on measured shared causes of death and dementia (that is, variables such as **C** in Figure 1). For an individual who has died by time *t*, the year-*t* censoring weight is zero. We estimated survival probabilities using a logistic regression model for death as a function of baseline and time-varying covariates. Baseline covariates included smoking status, age, sex, apolipoprotein E ε4 status, and educational attainment; time-varying covariates included systolic blood pressure, body mass index, and prevalent and incident comorbid heart disease, cancer, stroke, and diabetes. All modeling specifications and weights assessment are presented as Web Appendix 4. For illustrative purposes, we contrast how estimates change when relying on: 1) unconditional exchangeability for censoring by death; 2) exchangeability for censoring by death conditional only on baseline covariates; and 3) exchangeability for censoring by death conditional on both baseline and time-varying covariates (main results) in the Web Table 2.

We also estimated the total effect of smoking on mortality risk applying the Kaplan-Meier estimator with the weights calculated for handling confounding. We therefore assume that the same set of measured confounders used to estimate the total effect of smoking on dementia risk are sufficient for addressing confounding of the total effect of smoking on mortality risk. Estimates of all effects at 20 years of follow-up are presented as risk differences and risk ratios. All 95% confidence intervals were calculated using percentile-based bootstrapping with 500 bootstrap samples.

## RESULTS

Of 10,994 Rotterdam Study participants, 4,179 individuals met eligibility criteria (55–70 years, reported smoking history at baseline, and did not have history of dementia at study entry). The mean age was 62 years, and 1,870 (44.7%) were women (Table 2). In total, 368 (8.8%) developed dementia and 1,318 (31.5%) died over 20 years of follow-up. The median time to dementia was 15.5 years, and the median time to death was 13.1 years. Overall, from 1,572 who were current smokers at baseline, 117 (7.4%) developed dementia and 630 (40.1%) died; of the 2,607 former smokers, 251 (9.6%) developed dementia and 688 (26.4%) died.

**Table 2:** Descriptive Characteristics of Former and Current Smokers in the Rotterdam Study, the Netherlands, 1990–2015

	**Former Smokers(*n* = 2,607)**		**Current Smokers(*n* = 1,572)**	
**Characteristic**	**No.**	**%**	**No**	**%**
Age, years^a^	62.35 (4.0)		61.69 (4.0)	
Women	1,090	41.8	780	49.6
Education				
Primary education	258	9.9	198	12.6
Lower or intermediate general education or lower vocational education	1,080	41.4	693	44.1
Intermediate vocational education or higher general education	862	33.1	483	30.7
Higher vocational education or university	399	15.3	190	12.1
Unknown	8	0.3	8	0.5
APOE-ε4, carrier status				
Noncarrier	1747	67	1,074	68.3
1 allele	687	26.4	380	24.2
2 alleles	71	2.7	33	2.1
Unknown	102	3.9	85	5.4
Systolic blood pressure, mm Hg^a^	137.59 (20.8)		135.22 (21.3)	
Body mass index^a^^,^^b^	26.93 (3.7)		25.86 (3.8)	
Prevalent hypertension diagnosis	1,468	56.3	767	48.8
Prevalent stroke	52	2	23	1.5
Prevalent heart disease diagnosis	226	8.7	72	4.6
Unknown heart disease diagnosis	42	1.6	28	1.8
Prevalent diabetes diagnosis	275	10.5	147	9.4
Unknown diabetes diagnosis	389	14.9	364	23.2
Prevalent cancer diagnosis	69	2.6	27	1.7

Abbreviation: APOE, apolipoprotein E.

^a^ Values are expressed as mean (standard deviation).

^b^ Weight (kg)/height (m)^2^.

**Figure 2:**
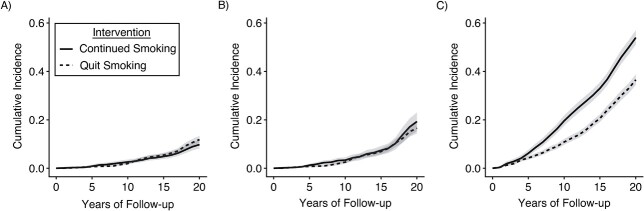
Risk of dementia and death by smoking cessation status over 20 years of follow-up, using data from the Rotterdam Study, the Netherlands, 1990–2015. A) Total effect on dementia risk: Curves represent the cause-specific cumulative incidence or crude risk of dementia over 20 years of follow-up had participants continued smoking vs. quit smoking. B) Controlled direct effect on dementia risk: Curves represent the marginal cumulative incidence or net risk of dementia (had death been eliminated) over 20 years of follow-up had participants continued smoking vs. quit smoking. C) Total effect on mortality: Curves represent the cumulative incidence or risk of all-cause mortality over 20 years of follow-up had participants continued smoking vs. quit smoking.

We estimated a total effect of smoking cessation (compared with continued smoking) on 20-year dementia risk of 2.1 (95% confidence interval: −0.1, 4.2) percentage points (Table 3; Figure 2). This slightly harmful effect estimate of quitting smoking (with wide confidence intervals) includes all causal pathways, including those through death. The presence of these pathways (from smoking to death) is clear from decades of research but also evidenced in these data: The estimated total effect of quitting smoking on 20-year mortality risk was –17.4 (95% confidence interval: −20.5, −14.2) percentage points. Alternatively, we estimated a controlled direct effect of quitting smoking on 20-year dementia risk, had death been fully prevented during the study period, as −2.7 (95% confidence interval: −6.1, 0.8) percentage points.

**Table 3:** Total Effect and Controlled Direct Effect of Smoking Cessation (Compared With Continued Smoking) on the Risk of Dementia, and the Total Effect on Risk of Mortality, at 20 Years of Follow-up, for Participants From the Rotterdam Study, the Netherlands, 1990–2015

**Causal Effect**	**Risk Difference**	**95% CI**	**Risk Ratio**	**95% CI**
Total effect on dementia	2.1	−0.1, 4.2	1.21	0.99, 1.50
Controlled direct effect on dementia (with IPCW for death)	−2.7	−6.1, 0.8	0.86	0.72, 1.05
Total effect on mortality	−17.4	−20.5, −14.2	0.68	0.63, 0.72

Abbreviations: CI, confidence interval; IPCW, inverse-probability-of-censoring weights.

## DISCUSSION

In longitudinal (randomized and observational) studies where dementia is the main outcome and deaths occur during follow-up, having clear causal questions and being explicit about the assumptions required for answering them will lead to more transparent interpretation of results, more appropriate analytical solutions, and a deeper understanding about plausible sources and magnitudes of bias. We considered 2 causal questions, beginning with the total effect, which captures all causal pathways including those mediated by death. In our example, the small estimated harmful total effect of smoking cessation on dementia risk necessarily captures some “protection” against dementia by death. This is not a “bias” but rather a feature of the total effect as the research question.

The controlled direct effect does not have this feature, and in our example, we estimated a small reduction in dementia risk if death was eliminated. However, residual bias from failing to adjust for a sufficient set of shared causes of death and dementia can remain, especially if only baseline covariates are considered (as observed in results of Web Table 2). Because exchangeability for censoring cannot be verified empirically, deep knowledge of the biological process and data generation mechanisms are needed (2). Furthermore, upper and lower bounds for extreme scenarios of dependency between the outcome of interest and the competing event can be estimated for additional understanding (2, 6, 39, 54). Since the controlled direct effect refers to a fictional scenario where everyone remains alive, the interpretation of this estimand still remains vague given that how to achieve such a scenario is not clear (55–57). We focused on this notion of direct effect as an alternative to the total effect to drive analysis; as we discussed, it can be estimated using popular and familiar analytical approaches in the survival analysis literature.

Alternative questions can be posed when competing events are present, some also capturing notions of direct effect. For example, the “survivor average treatment effect” quantifies the effect of a treatment on a subgroup of individuals who would not die during the study period under either level of treatment (32, 58). However, the utility of this question is questionable as this subgroup is not observable and may not even exist. The novel alternative of “separable effects” avoids evoking scenarios that “eliminate death” or unobservable subpopulations (31, 33). Separable effects are effects of modified treatments motivated by the physical decomposition of the exposure assumed to operate on dementia and death through separate pathways or completely different treatments that operate like the study treatment. Statistics for these estimands do not coincide with “off-the-shelf” survival analysis methods and at this time require more novel software (59). Of course, yet another notion of effect in competing event settings is the total effect on the combined outcome endpoint, such as the effect on dementia or death. This is simply a combination of the total effects on dementia and death separately and will only lose information compared with targeting these separate total effects (8). In our example, because the total effect of smoking on mortality is so large, it would drive the magnitude of this composite effect.

Too often, we start by choosing a statistical method without clarification on the research question of interest. In a setting with competing events, choosing a research question is not straightforward. In this work, we focused on 2 possible causal questions that can lead to familiar statistical procedures as an accessible step forward beyond the use of Cox proportional hazards analysis. Through our discussion and application, we hope that readers will see an opportunity to reconceptualize how to ask clearer questions in the context of competing events, and let the questions lead toward the methods that best suit the aim.

## Supplementary Material

Web_Material_kwad090
