# Supplementary material for: Considering Questions Before Methods in Dementia Research With Competing Events and Causal Goals
Source: Am J Epidemiol. 2023 May 3;192(8):1415–23. doi: 10.1093/aje/kwad090 (PMC10403306; doi:10.1093/aje/kwad090)
Supplement: Web_Material_kwad090 [file web_material_kwad090.pdf]

Web Materia

Considering Questions Before Methods in Dementia Research With Competing Events and Causal Goals

L. Paloma Rojas-Saunero; Jessica G. Young; Vanessa Didelez; M. Arfan Ikram; Sonja A. Swanson

Table of contents:

Web Appendix 1. Systematic Review on reporting of longitudinal studies focused on exposure effects on dementia risk 2

Web Figure 1. Flowchart of paper selection for systematic review 3

Web Table 1. Current reporting practices relevant to competing events in dementia research among 57 studies included in the systematic review 4

Web Appendix 2. Identifiability assumptions for the total and direct effect 5

Web Appendix 3. The Rotterdam Study, outcome assessments 7

Web Appendix 4. Modelling specifications for inverse probability weighting 8

Web Table 2. Controlled direct effect of smoking cessation (compared to continued smoking) on the risk of dementia at 20 years of follow-up, evoking alternative exchangeability assumptions for censoring by death 9

References 10

Web Appendix 1. Systematic Review on reporting of longitudinal studies focused on exposure effects on dementia risk

We performed a systematic review of longitudinal studies focused (implicitly or explicitly) on exposure effects on dementia risk, in order to summarize how death during follow-up is handled in the design, analysis, reporting, and interpretation of results.

Eligibility for the systematic review included: original research with longitudinal data on dementia or Alzheimer's disease outcomes; published between January 2018 to December 2019; published in one of nine medicine or neurology journals; and having an implicit or explicit study aim of estimating a causal effect. Search criteria was defined as follows:

(("Neurology"[Journal] OR "JAMA"[Journal] OR "JAMA neurology"[Journal] OR "lancet london england"[Journal] OR "the lancet neurology"[Journal] OR "Annals of neurology"[Journal] OR "alzheimer s dementia the journal of the alzheimer s association"[Journal] OR "The New England journal of medicine"[Journal] OR "bmj clinical research ed"[Journal]) AND (("alzheimer disease"[MeSH Major Topic] OR "dementia"[All Fields]) AND ("longitudinal"[All Fields] OR "longitudinally"[All Fields] OR "cohort studies"[MeSH Terms] OR "cohort"[All Fields]) AND ("hazard"[All Fields] OR "hazard s"[All Fields] OR "hazardous"[All Fields] OR "hazardously"[All Fields] OR "hazardousness"[All Fields] OR "hazards"[All Fields] OR ("risk"[MeSH Terms] OR "risk"[All Fields]))) AND (2018:2019[pdat])

Eligibility criteria included:

- Original research
- Study design corresponds to an observational study or randomized trial with longitudinal follow-up
- Implicitly or explicitly interested in estimating a causal effect, such that:
    - A clear definition of one or more exposures, interventions, or treatments and,
    - is not aimed at describing the prevalence or incidence of dementia (i.e., a clearly descriptive aim) and,
    - is not aimed at building or validating a prediction model or assessing diagnostic testing accuracy of a biomarker or proxy for dementia diagnosis (i.e., a clearly predictive aim) and,
    - uses methods to handle confounding, or
    - discusses conclusions or implications about results that are causal (such as that they unveil mechanisms, potential targets of intervention, change clinical practice or guide public health decisions)
- The outcome of interest is time to incident case of dementia, Alzheimer's disease or cognitive impairment

We collected the following information: study characteristics (exposure of interest, target causal parameter, median length of follow-up); report on total deaths over time and across levels of the exposure of interest and total losses to follow-up; specific methodologic considerations (how death is handled in the analysis plan, primary statistical measure, primary statistical method); and interpretation (valid interpretation of the primary result in light of deaths, mentions mortality in discussion).

Fifty-seven studies ultimately met our eligibility criteria (**Web Figure 1)** Mean or median follow-up was over 5 years for 84% of the studies (**Web Table 1**). The number or proportion of individuals who died over time was reported in 56% of papers; 18% presented these numbers by exposure level. Only 11% had a clear and complete description of how death was treated in the main analysis, while 47% did not include any description on how death was handled in the methods section. The vast majority (93%) presented estimates of a hazard ratio, mostly under a Cox proportional hazards model though

none reported the correct interpretation given the presence of a competing event nor discussed the assumptions related to death as a competing event. Furthermore, 86% interpreted hazard ratios as inferring something about a risk (e.g. “the exposure increased the risk of dementia, HR:X, 95%CI”) and only one study gave an explicit interpretation that matched the target causal parameter of interest. Overall, only one-third mentioned death in some context in the discussion section.

Web Figure 1. Flowchart of paper selection for systematic review

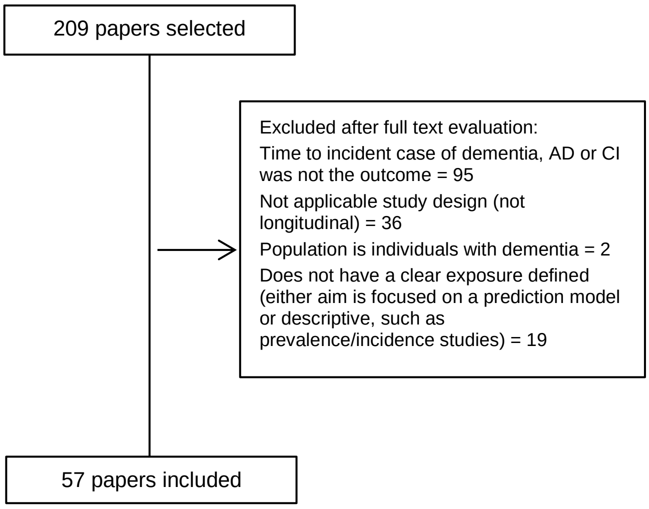

Web Table 1. Current reporting practices relevant to competing events in dementia research among 57 studies included in the systematic review

	N (%)
Exposure type	
Time-fixed or time-varying measured at one time point	45 (79%)
Time-varying	3 (5%)
Time-varying treated as time-fixed	9 (16%)
Target causal parameter	
Risk difference without elimination of death	1 (2%)
Unclear or not stated	56 (98%)
Median length of follow-up	
1 to 3 years	0
3 to 5 years	9 (16%)
5 to 10 years	20 (35%)
10 to 15 years	9 (16%)
15 to 20 years	5 (6%)
Above 20 years	14 (25%)
Includes number or percentage of deaths	32 (56%)
Includes number or percentage of loss to follow-up	32 (56%)
Includes number or percentage of mortality by exposure level	10 (18%)
Information on how the competing event of death is handled in the analysis plan	
Does not include any description of how the event of death was defined	27 (47%)
Only defined death as a censoring event	8 (14%)
Defines the event of death as part of a sensitivity analysis	15 (26%)
Defines the event of death as part of the main analysis with clear description of the methods/assumptions for valid estimation	6 (11%)
Unclear description	1 (2%)
Primary statistical method	
Cause-specific hazard model	51 (89%)
Cumulative incidence function	1 (2%)
Fine-Gray sub distribution hazard model	2 (4%)
Poisson model	1 (2%)
Other	2 (4%)
Primary statistical measure	
Cause-specific hazard ratios	53 (93%)
Risk Ratios	2 (4%)
Cumulative risks (absolute risk - risk difference)	1 (1%)
Sub-distribution hazard ratios	1 (1%)
Interpretation of the primary estimate given the competing event of death	
No interpretation given	4 (7%)
Only interprets null hypothesis test	3 (5%)
Potentially incomplete/inaccurate interpretation	49 (86%)
Interpretation is explicitly defined as the target causal parameter	1 (2%)
Mentions mortality in discussion section	18 (32%)

Abbreviation: N, number of articles

Web Appendix 2. Identifiability assumptions for the total and direct effect

In this section, we consider assumptions under which the total effect and controlled direct effect under elimination of competing events can be identified in a study where exposure is randomized at baseline or an observational study like the Rotterdam Study where there is no loss to follow-up. For additional details see Young et al(1).

Let A be an indicator of exposure (A = 1 for individuals who stopped smoking at baseline, A = 0 for those who continued at baseline). Let k = 0,…. K +1 denote equally spaced follow-up annual intervals with k = 0 corresponding to baseline and k = K + 1 to the maximum follow-up time of interest, in this case 20 years post baseline. Let Y_k and D_k denote indicators of dementia and death by interval k, respectively. By definition $D_0 \equiv Y_0 \equiv 0$ because the study population is restricted to those who have not yet experienced dementia diagnosis and are alive at baseline. For k > 0, let L_k denote a vector of time-varying individual characteristics updated by k (e.g, systolic blood pressure, BMI) with baseline covariates L_0 (e.g. age, sex, APOE ε4 status, and educational attainment) measured before assignment to treatment A. We denote the history of a random variable using overbars, for example, $\bar{Y}_k = (Y_0, \dots, Y_k)$ is the history of the event of interest through interval k. We denote the future of a random variable through the follow-up of interest using underbars, for example, $\underline{Y}_{k+1} = (Y_{k+1}, \dots, Y_{K+1})$. Given the nature of how dementia diagnosis was measured, there is no loss to follow-up in this study. For simplicity, we assume that all variables where measured without measurement error.

Total effect:

To identify the risk of dementia and in turn the total effect, we must make untestable assumptions. Specifically, for both $a = 1$ and $a = 0$, and for each k = 0,…, K consider the following three identifying assumptions:

1. Exchangeability:

$$\bar{Y}^a_{k+1} \coprod A | L_0,$$

This assumption requires that baseline observed treatment, conditional on the measured past is independent of future counterfactual outcomes had everyone followed $A = a$.

2. Positivity:

$$f_{L_0}(l_0) > 0 \Rightarrow$$

$$\Pr[A = a | L_0 = l_0] > 0$$

Where $f_{L_0}(l_0)$ is the joint density of L_0 evaluated at l_0.

3. Consistency

If $A = a$,
then $\bar{D}^a_{k+1}$ and $\bar{Y}_{k+1} = \bar{Y}^a_{k+1}$

This assumption requires well-defined interventions such as that, if an individual has data consistent with the interventions indexing the counterfactuals outcome through k + 1, then the observed

covariates and outcomes through k + 1 equal the counterfactual outcomes and covariates under that intervention. These 3 assumptions are guaranteed in a study where A is physically randomized within levels of L_0 but are not guaranteed in an observational study like the Rotterdam Study.

Direct effect:

To identify the risk of dementia under elimination of death, and in turn the controlled direct effect, we must make untestable assumptions that are stronger than those required for the total effect. These are not jointly guaranteed even in a study where A is physically randomized within levels of L_0.

For each k = 0,…, K consider the following three identifying assumptions.

1. Exchangeability

$$\underline{Y}_{k+1}^{a,\bar{d}=0} \coprod A | L_0,$$

$$\underline{Y}_{k+1}^{a,\bar{d}=0} \coprod D_{k+1} | \bar{L}_k = \bar{l}_k, \bar{Y}_k = \bar{D}_k = 0, A = a$$

Where $\bar{l}_k$ is some realization of $\bar{L}_k$. This assumption requires that baseline observed treatment, conditional on the measured past is independent of future counterfactual outcomes.

In addition, it requires that at each follow-up time, conditional on past survival, not having yet developed dementia and past values of measured covariates, whether an individual dies is independent of future counterfactual outcomes under $A = a$ and elimination of death. Because the competing event cannot be randomly assigned (or prevented) by an investigator in practice, this condition will not hold by design even in an experiment where A is randomized.

2. Positivity:

In addition to the positivity assumption for the total effect, we require the following:

$$f_{A,\bar{L}_k,D_k,Y_k}\left(a, \bar{l}_k, 0,0\right) \neq 0 \Rightarrow$$

$$\Pr[D_{k+1} = 0 | \bar{L}_k = \bar{l}_k, D_k = Y_k = 0, A = a] > 0$$

Where $f_{A,\bar{L}_k,D_k,Y_k}\left(a, \bar{l}_k, 0,0\right)$ is the joint density of $(A, \bar{L}_k, D_k, Y_k)$ evaluated at $\left(a, \bar{l}_k, 0,0\right)$. This assumption requires that, for any possibly observed level of treatment and covariate history amongst those remaining uncensored (alive) and free of dementia diagnosis through k, some individuals continue to remain alive through k + 1.

3. Consistency

If $A = a$ and $\bar{D}_{k+1} = 0$,
then $\bar{L}_{k+1} = \bar{L}_{k+1}^{a,\bar{d}=0}$ and $\bar{Y}_{k+1} = \bar{Y}_{k+1}^{a,\bar{d}=0}$

This assumption requires that, if an individual has data consistent with the interventions indexing the counterfactuals outcome through k + 1, then the observed outcomes and covariates through k + 1 equal the counterfactual outcomes and covariates under that intervention. The consistency assumption requires well-defined interventions, which is problematic in this scenario because the estimand implies an unspecified intervention to eliminate death prior to dementia diagnosis.

Web Appendix 3. The Rotterdam Study, outcome assessments

Dementia diagnosis: Diagnosis was collected by screening during the five study visits, using MMSE and the Geriatric Mental Schedule (GMS) organic level. Screen-positives (MMSE<26 or GMS organic level>0) subsequently underwent an examination and informant interview with the Cambridge Examination for Mental Disorders in the Elderly. A consensus panel led by a consultant neurologist established the final diagnosis according to standard criteria for dementia (DSM-III-R). Additionally, participants were continuously followed for the occurrence of dementia through automated linkage of the study database and digitized medical records from general practitioners and the Regional Institute for Outpatient Mental Health Care. For participants who moved outside the study district or lived in nursing homes, medical records were regularly checked by contacting their treating physicians. Research physicians reviewed all potential dementia cases using hospital discharge letters and information from general practitioners and nursing home physicians (2). Linkage-based diagnoses were based on data up through December 2015.

Vital status: Vital status was obtained on a weekly basis via municipal population registries and through general practitioners' and hospitals' databases, with complete linkage up through December 2015.

Web Appendix 4. Modelling specifications for inverse probability weighting

- **Treatment weight denominator model**

Dependent variable: smoking (0 = current, 1 = former)

Independent variables: age at study entry with natural cubic splines, sex (women vs. men), education (five categories), APOE-ε4 (four categories), cohort (two categories), and no product terms between covariates

- **Censoring by death weight denominator model:**

Dependent variable: death status at year k (0 = no, 1 = yes)

Independent variables: smoking (0 = current, 1 = former); death dementia diagnosis (0 = no, 1 = yes); year t with natural cubic splines; age at study entry with cubic splines, sex (women vs. men), education (five categories), APOE-ε4 (four categories), cohort (two categories), prevalent diabetes (yes, no), baseline blood pressure with cubic splines, baseline BMI with cubic splines, prevalent hypertension (yes, no); indicator for incident cancer (yes, no), incident heart disease (yes, no), incident diabetes (yes, no) and incident stroke (yes, no) and no product terms between covariates.

Web Table 2. Controlled direct effect of smoking cessation (compared to continued smoking) on the risk of dementia at 20 years of follow-up, evoking alternative exchangeability assumptions for censoring by death

	Risk of dementia, smoking cessation arm	Risk of dementia, continued smoking arm	Causal Risk Difference (95%CI)	Causal Risk Ratio (95%CI)
**Evoking unconditional exchangeability assumption for censoring***	16.2 (13.9, 18.3)	15.5 (13.9, 16.9)	-0.70 (-3.3, 2.2)	0.96 (0.82, 1.16)
**Evoking conditional exchangeability assumption on baseline covariates for censoring +**	18.0 (15.4, 20.4)	16.5 (14.9, 18.0)	-1.53 (-4.6, 1.8)	0.92 (0.78, 1.12)
**Evoking conditional exchangeability assumption on baseline and time-varying covariates for censoring++**	19.3 (16.1, 21.2)	16.5 (14.9, 18.3)	-2.75 (-6.1, 0.8)	0.86 (0.7, 1.1)

Abbreviation: IPCW, Inverse probability censoring weights

* Without IPCW

+ Baseline covariates include: age at study entry with cubic splines, sex (women vs. men), education (five categories), APOE-ε4 (four categories), cohort (two categories)

++ Time-varying covariates include: age at study entry with cubic splines, sex (women vs. men), education (five categories), APOE-ε4 (four categories), cohort (two categories), prevalent diabetes (yes, no), baseline blood pressure with cubic splines, baseline BMI with cubic splines, prevalent hypertension (yes, no); indicator for incident cancer (yes, no), incident heart disease (yes, no), incident diabetes (yes, no) and incident stroke (yes, no) and no product terms between covariates

References

1. Young JG, Stensrud MJ, Tchetgen Tchetgen EJ, et al. A Causal Framework for Classical Statistical Estimands in Failure-time Settings with Competing Events. *Statistics in Medicine*. 2020;39(8):1199–1236.

2. de Bruijn RFAG, Bos MJ, Portegies MLP, et al. The potential for prevention of dementia across two decades: The prospective, population-based Rotterdam Study. *BMC Medicine*. 2015;13(1):1–8.
